# Bioeffects of *Prunus spinosa* L. fruit ethanol extract on reproduction and phenotypic plasticity of *Trichoplax adhaerens* Schulze, 1883 (Placozoa)

**DOI:** 10.7717/peerj.6789

**Published:** 2019-04-18

**Authors:** Maria Cristina Albertini, Daniele Fraternale, Federica Semprucci, Silvio Cecchini, Mariastella Colomba, Marco B.L. Rocchi, Davide Sisti, Barbara Di Giacomo, Michele Mari, Luigia Sabatini, Lucia Cesaroni, Maria Balsamo, Loretta Guidi

**Affiliations:** Department of Biomolecular Sciences, University of Urbino, Urbino, Pesaro-Urbino, Italia

**Keywords:** *Prunus spinosa*, Bioactive compounds, Food supplement, *Trichoplax adhaerens*, Ethanol damage

## Abstract

The aim of this work was to test and analyse the bioeffects of *Prunus spinosa* L. (Rosacaee) fruit ethanol extract on *Trichoplax adhaerens* Schulze, 1883 (Placozoa) laboratory cultures which—for the first time—were employed as *in vivo* biological model to assess the bioactivity of a natural extract. The ethanol extract of *P. spinosa* was administrated during a 46 day experimental period; ultrastructural (by optical, confocal, TEM and SEM microscopy) and morphometric analyses indicated that treated *Trichoplax adhaerens* showed significant differences in viability, reproductive modalities, body shape and colour with respect to the control group. Finally, *P. spinosa* bioactive compounds seem to exert profound protective effects on *T. adhaerens* reproduction and phenotype. Our results may support additional investigations related to other bioactive compounds properties useful for nutraceutical preparations to be used as food supplements.

## Introduction

Medicinal plants are the richest and primary sources of natural bioactive compounds used in traditional and modern medicine. For this reason, they are attracting growing interest as constituents of natural products useful in prevention and adjunctive therapies of multiple chronic diseases. One such plant is *Prunus spinosa* L. (blackthorn), a wild plum shrub/small tree belonging to the Rosaceae family. Native to Scotland, it is commonly found in Europe growing on slopes of wide uncultivated areas, and in deciduous forests and temperate countries in Asia (Kumarasamy et al., 2003). *Prunus spinosa* fruits contain a combination of bioactive phytochemicals and nutrients (proteins 2.86 g/100 g of dry weight and fats 1.98 g/100 g dry weight), which make them a potential source of nutraceuticals (Barros et al., 2010). Nevertheless—due to their unpleasant tart taste—they are not consumed directly as human food, with the only exception of the fruit juice commonly used as flavouring in liqueurs and wine (Fraternale et al., 2009a) and in formulation of new isotonic drinks (Gironés-Vilaplana et al., 2014).

For medicinal applications, the plant has been used throughout Europe, with the flowers being the most popular in central and eastern parts of the continent thanks to its purgative, diuretic and detoxifying properties. The fruits, on the other hand, are used in different preparations of natural medicines indicated mainly in mild inflammation of the oral and pharyngeal mucosa, as well as an astringent (Aparajita, Juan Pedro & Itziar, 2002; Marchelak et al., 2017).

*P. spinosa* major phytochemicals are flavonoids, in particular anthocyanins, which are responsible for the blue and purple colour (Deineka, Deineka & Sirotin, 2005) and, in addition, are supposed to play a very important role in antioxidant processes. Anthocyanins, in fact, represent one of the main classes of water-soluble flavonoids that are able to influence different enzymatic activities and gene expression through their effects on nuclear receptors (Avior et al., 2013).

We have previously demonstrated antioxidant activity of fresh *P. spinosa* fruit juice and its biological activity (cytoprotective activity) in cultured human promonocytes (U937 cells) exposed to hydrogen peroxide (Fraternale et al., 2009b). Recently, cyanidin-3-o-β-glucoside has also been demonstrated to have an inhibitory effect on nuclear factor kappa B (NF-kB) activation (Ya et al., 2018). The fruits of *P. spinosa* also contain carotenoids, mostly β-carotene and lutein (Mikulic-Petkovsek et al., 2016), which are able to modulate several molecular mechanisms (Milani et al., 2016). However, despite all these promising results, the potential of blackthorn as a source of bioactive extracts still remains only partially explored. Therefore, this study was performed to characterize *P. spinosa* fruit extract with respect to its biological activity by *in vivo* experiments carried out on *Trichoplax adhaerens* Schulze, 1883—a new model organism recently used in human biomedical and biological studies (Von der Chevallerie, Rolfes & Schierwater, 2014; Schierwater & DeSalle, 2018) and now employed for the first time in experiments concerning natural extracts bioactivity assessment.

*T. adhaerens* Schulze, 1883—one of the three formally described Placozoa species (along with *Hoilungia hongkongensis* Eitel, Schierwater, et Wörheide, 2018 and Polyplacotoma mediterranea Osigus & Schierwater, 2019)—are non-bilaterian marine animals with the simplest morphology among all the metazoans, consisting of two epithelial layers separated by a tridimensional network of star-shaped fibers (Grell, 1972; Ivanov, 1973; Behrendt & Ruthmann, 1986; Ruthmann, Behrendt & Wahl, 1986; Grell & Ruthmann, 1991; Buchholz, 1995). These organisms are flat, with irregular margins, without manifest body symmetry or tissues, the only form of polarity being the distinction between the lower surface (facing the substrate) and the upper surface (facing the open water). Placozoans possess only seven different somatic cell types: lower epithelial cells, upper epithelial cells, gland cells, fiber cells, lipophil cells, crystal cells and small potentially “omnipotent” stem cells. These small cells are located in a ring around the periphery of *Trichoplax,* at the contact point of the upper and lower epithelium (Guidi et al., 2011; Smith et al., 2014; Schierwater & Eitel, 2015). Although the two cell layers are reported as epithelial layers, neither the basal lamina nor the extracellular matrix are present, a condition known only for Placozoa (Schierwater, 2005). Standard reproduction is by binary fission (Grell, 1984), whereas budding occurs only when environmental conditions become unfavourable and produces small planktonic spherical swarmers, which represent dispersal stages (Thiemann & Ruthmann, 1988; Thiemann & Ruthmann, 1991). Sexual reproduction has been shown to occur in nature (Signorovitch, Dellaporta & Buss, 2005) and has been induced too under laboratory conditions. However, the embryos from such matings develop only to the 128-cell stage and then die (Grell, 1971; Grell, 1972; Grell, 1984; Signorovitch, Buss & Dellaporta, 2007; Eitel & Schierwater, 2010).

Despite the simple organization of these organisms and the lack of synapses, they may show complex coordinated behaviours, such as positive phototaxis (Srivastava et al., 2008). A complex sequence during feeding activities culminating in external digestion of microalgae has also been observed (Smith, Pivovarova & Reese, 2015). In detail, they form a digestive cavity lifting up the central body region between the substrate and the lower epithelium (Schierwater, 2013; Schierwater & Eitel, 2015). The gland and lipophil cells are the two types of digestive cells in *Trichoplax* that secrete granular material able to lyse food source (Smith, Pivovarova & Reese, 2015). Food may also be trapped by the upper epithelial cells and phagocytized by the extensions of the fiber cells through the so-called “transepithelial cytophagy” (Schierwater, 2013).

In laboratory cultures, *T. adhaerens* may change some morphological features, such as body shape, size, colour, transparency, even growth and reproduction modalities, in relation to the different types of microalgae used as food (e.g., *Chlorella* sp. or *Rhodomonas salina*). These changes could be the results of different algal pigments interacting with nuclear receptors (NRs) and, indeed, analysis of NRs in differently sized animals indicates impact of food composition on NR expression (Novotný et al., 2017).

For all these reasons, taking into account that *T. adhaerens* represents a novel model with numerous potentialities in biological and biomedical research (i.e., the simplest bauplans of all metazoans; the smallest nuclear genome and the largest mitochondrial animal genome; the occurrence of representatives of all major gene families present in humans; and easiness in maintaining laboratory cultures) (Signorovitch, Dellaporta & Buss, 2006; Schierwater, De Jong & DeSalle, 2009; Schierwater et al., 2009; Eitel et al., 2013; Schierwater et al., 2016; Schierwater & DeSalle, 2018), the biological activity of *P. spinosa* fruit ethanol extract was investigated by *in vivo* tests in order to evaluate reproduction modality, phenotype variability and feeding behaviour of *T. adhaerens* in response to the administration of the extract.

## Materials & Methods

### Ethanol extract of *Prunus spinosa*

Fully ripe fruits (10–15 mm of diameter) of *P. spinosa* L. subsp. *spinosa* growing spontaneously in a circumscribed territory (Urbino, the Marches, central Italy, at 500 m a.s.l., GPS coordinates N43°40′46.512″–E12°31′42.291″) were used for the present study.

According to the basic procedure of extraction of natural bioactive compounds by ethanol concentrated (to achieve the highest concentration of biocomponents) solutions subsequently diluted to working solutions, the ethanol extract of *P. spinosa* was prepared as follows: frozen *P. spinosa* fruits without kernels (50 g) were homogenized in 50 mL of 70% ethanol solution and centrifuged at 10,000 *g* for 15 min. The supernatant was collected and the residue was re-extracted under the same conditions. The two supernatants were reunited and concentrated up to 50 mL by rotary evaporation at 37 °C. After evaporation, dried extract was weighted and resuspended in a 70% ethanol solution to obtain a 100x solution (4 mg/mL) to be used at the final concentration of 40 µg/mL.

### Cultures of *Trichoplax adhaerens*

The “Grell” clone used for this study belong to laboratory cultures reared for many years at the Urbino University and coming from original specimens collected by Eitel & Schierwater (2010). Clones were maintained in Duran glass Petri dishes (10 mm grid, 10 × 2 cm) filled with artificial seawater (Reef Crystals® Reef Salt, salinity 30-33 PSU) supplemented with soil extract (http://www.epsag-uni-goettingen.de), KNO_3_ (0.2 g/L), K_2_HPO_4_ (20 mg/L) and Mg_2_S0_4_(20 mg/L) at 20–22 °C. Petri dishes were kept in a covered moist chamber to avoid evaporation and *Chlorella salina* (Chlorophyta, Chlorophyceae) was added as food. In all Petri dishes, several coverslips (VWR, 18 × 18 mm) were placed, on which animals tended to move; coverslips were then used for transferring specimens to other Petri dishes so to increase the number of cultures. *Chlorella salina*—from the Culture Collection of Algae at Göttingen University (http://www.epsag-uni-goettingen.de)—was kept in Duran glass flasks (250 mL) filled with artificial seawater (see above) at room temperature (20–22 °C) and added to the Petri dishes every 5 days. The flasks were maintained with no natural light, 1 m distant from 2 artificial lamps (Osram L 36 W/77 Fluora and OSRAM L36 W/965 Biolux) with a 12:12 h light:dark cycle. Guillard’s (F/2 Sigma-Aldrich) marine enrichment medium was added to the cultures at the concentration of 2%. When the algal cultures reached an appropriate density, 50% of the content of several flasks was transferred in centrifuge tubes and centrifuged (15 min at 410 *g*). Once removed the supernatant, the algal concentrate was poured into 1.5 ml Eppendorf tubes, which were frozen at −18 °C.

For this study, three subcultures were set. Each subculture was started by putting a coverslip with at least three specimens on top into a new Petri dishe filled with artificial seawater. To avoid a carpet of alive algae interfering with animals counting, defrozen algae were resuspended in artificial seawater, and added to Petri dishes (100 µl each) as food. The three subcultures were considered running after two weeks, when at least eight specimens were found on the coverslip.

### Experimental protocol

Three different treatment groups have been set up from the three subcultures. The control group (CTRL) was untreated, the second group (labelled as EtOH) was treated with Ethanol only (vehicle, 0.7% EtOH) and the third group (indicated as P + EtOH) was treated with *P. spinosa* ethanol extract (*P. spinosa* extract 40 µg/mL + 0.7% EtOH). Ethanol and *Prunus* ethanol extract solutions were added only once (to avoid changes in final ethanol concentration) after the two weeks that were necessary to start the subcultures. Observations were made under stereomicroscope (Nikon SMZ645 equipped with: eye piece 10x c-W10X; illuminator C-DSLS 6V-20W) every two days and animal visual counting performed every 2–5 days in each complete Petri dish. Our experimental protocol was planned for a total duration of 60 days (July 20–September 18, 2017; including 14 days for starting the cultures and 46 days for observation and counting) to investigate the *Prunus spinosa* ethanol effect over a medium-long period. At the end of the experiment (46th day), salinity was measured and resulted unchanged. Then, at least 20 living individuals were taken by a Pasteur pipette from each Petri dish, carefully transferred to a microscope slide observed under a Vanox AHBT3 Olympus optical microscope, photographed and recorded with a time-lapse video recorder (MikroCam 1.3 MP; Bresser). The morphometric analysis was carried out on photographed animals. Additional 20 living individuals were picked out from each treatment group and prepared for TEM analysis.

### Transmission Electron Microscopy (TEM) analysis

Specimens were fixed in 2% glutaraldehyde in 0.1 M sodium cacodylate buffer (pH 7.4) and stored in the same buffer until post fixation in 1% osmium tetroxide. After washing in sodium cacodylate buffer, the individuals were dehydrated in a graded ethanol series and embedded in Araldite. Semithin and ultrathin sections were cut using a glass knife and a diamond knife (Micro Star 3 mm), respectively with a LKB Ultrotome 2088V. Semithin sections were stained with toluidine blue and observed in transmission light under a Vanox AHBT3 Olympus optical microscope. Ultrathin sections were contrasted with uranyl acetate and lead citrate and observed with a Philips CM10 transmission electron microscope.

### Confocal Laser Microscope analysis

Ten living specimens were taken from the P+EtOH Petri dish after the 46th day, stained with Nile Red (Sigma-Aldrich) to localize neutral and polar lipids and observed *in vivo* under Zeiss LSM 510 Confocal Laser Microscope. Nile Red was prepared at 100 µg/ml in Dimethyl sulfoxide, and added to the culture medium for 15 min at a final concentration of 1 µg/ml. After incubation, samples were analysed; polar lipids (i.e., phospholipids) which are mostly present in membranes, were stained in red (emission  > 590 nm), whereas neutral lipids (esterified cholesterol and triglycerides), present in lipid droplets, were stained in yellow (570–590 nm).

### Scanning Electron Microscopy (SEM) analysis

At the end of the experimental period, ten individuals treated with *P. spinosa* fruit ethanol extract (P+EtOH) were prepared for scanning electronic microscopy (SEM) analysis. Specimens were fixed in 2% glutaraldehyde in 0.1 M sodium cacodylate buffer (pH 7.4), rinsed in the same buffer, dehydrated with a graded ethanol series, critical point-dried using CO_2_, mounted on aluminium stubs, sputter coated with gold palladium, and finally observed with a Philips 515 Scanning Electron Microscope.

### Growth analysis

Growth curve analysis was performed by a non-linear regression fitting a 4-parameter logistic growth curve with R^2^ multiple coefficient >0.75.

\begin{eqnarray*}{N}_{t}={N}_{0}+ \frac{{N}_{\max \nolimits }}{ \left( 1+{e}^{-1/b \left( t-c \right) } \right) } \end{eqnarray*}

where *t* is the measurement time, *N*_*t*_ is Placozoa number at cycle *t*, *N*_*max*_ is the maximal number expected, *c* is time of the turning point of the curve, *N*_0_ is the estimated initial number and *b* is the shape parameter.

The *Flex* inflection point coordinates (X_Flex_; Y_Flex_) were calculated as follows $:Flex= \left( c; \frac{{N}_{max}}{2} +{N}_{0} \right) $ and the tangent slope (m) in flex point was estimated as: $m= \frac{{N}_{max}}{4b} $. Non-linear regression was performed using Levenberg–Marquardt algorithm on SPSS 22.0 software. Multivariate analysis of variance (MANOVA) was performed in order to compare the groups. The significance level was set at *α* = 0.05.

### Principal Component Analysis (PCA)

To detect possible morphological differences among the groups subject to different treatments, a Principal Component Analysis (PCA) was performed on the morphometric variables detected in ImageJ software (https://imagej.nih.gov/ij/features.html). PCA allows to reduce the dimensionality of the data and to show a large number of variables in a two-dimensional plot. In our experimental setting, we had three groups of treatment. A diagram of the values obtained from each experimental group was plotted in the bi-dimensional space, defined by the 1st and 2nd Principal Component (PC1 and PC2, respectively) functions. The 95% confidence ellipses around group mean points are also reported. Multivariate analysis of variance (MANOVA) was performed in order to compare the groups. The significance level was set at *α* = 0.05. All the statistical analyses were performed with SPSS (Statistical Package for Social Sciences) release 23.0.

## Results

### Effects of *P. spinosa* fruit ethanol extract on *T. adhaerens* reproduction

**Figure 1:**
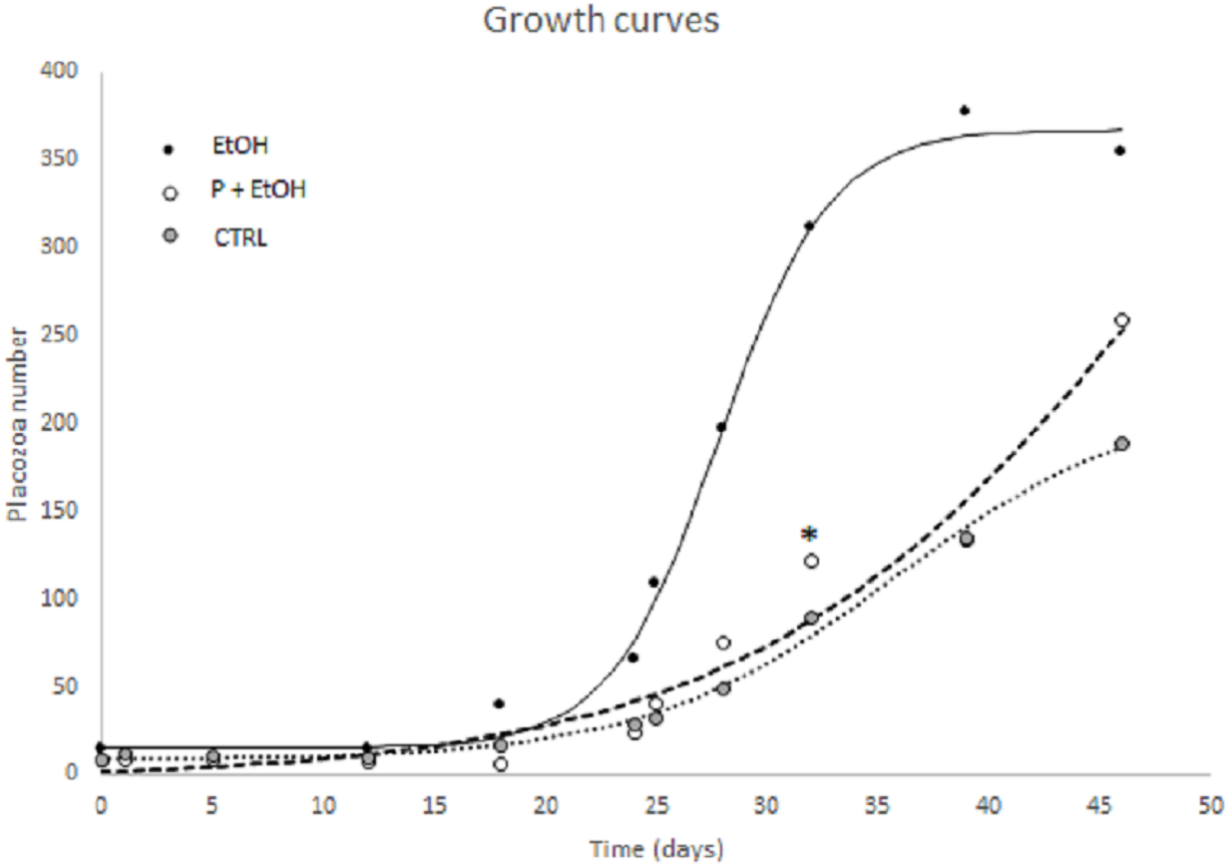
Non-linear logistic regression growth curve showing a significant goodness of fitting using a 4-parameter logistic curve. P+EtOH and CTRL groups were not significantly different, while the EtOH group resulted different from the other two experimental groups (*p* ≤ 0.05). *X* axis: culture days; *Y* axis: Placozoa number. Non linear logistic regression growth curve showing a significant goodness of fitting using a 4-parameter logistic curve. Animal counting was performed every 2–5 days in each complete Petri dish. P+EtOH and CTRL groups were not significantly different, while the EtOH group resulted different from the other two experimental groups (*p* ≤ 0.05). Asterisk shows that after 32 days of growth the P+EtOH group showed a number of animals higher than expected. *X* axis: culture days; *Y* axis: Placozoa number.

For each of three experimental conditions the logistic growth curve showed a significant goodness of fitting using a 4-parameter logistic curve. Comparing parameters (N_0_, N_max_, X_flex_, Y_flex_, m_flex_) obtained from non-linear regression, P+EtOH and CTRL groups were not significantly different, whereas the EtOH treated organisms resulted different (*p* ≤ 0.05) from the other two experimental groups. In detail, after nearly 34 days of growth the EtOH group showed a flex coinciding with time having a maximal growth (35.7 new Placozoa in a day, Table 1). This growth curve reached a plateau after about 40 days with a plateau value of 352.2 (estimated, see Table 1 and Fig. 1).

**Table 1:** Placozoa logistic growth curve. Comparing N_0_, N_max_, *X*_flex_, *Y*_flex_ and *m*_flex_, P+EtOH and CTRL groups were not significantly different, whereas the EtOH group was different from the other two experimental groups (bold values, *p* ≤ 0.05). The parameters obtained from non linear regression were the following: N_0_ (estimated initial number), N_max_ (maximal number expected), *X*_flex_ (*x* coordinate inflection point), *Y*_flex_ (*y* coordinate inflection point), *m*_flex_ (time of maximal growth).

	EtOH	P+EtOH	CTRL
N_0_	15.2	−2.8	8.3
N_max_	352.2	662.7	207.8
X_flex_	42.8	65.6	50.8
Y_flex_	191.3	328.6	112.2
m_flex_	35.7	16.3	9.1

**Figure 2:**
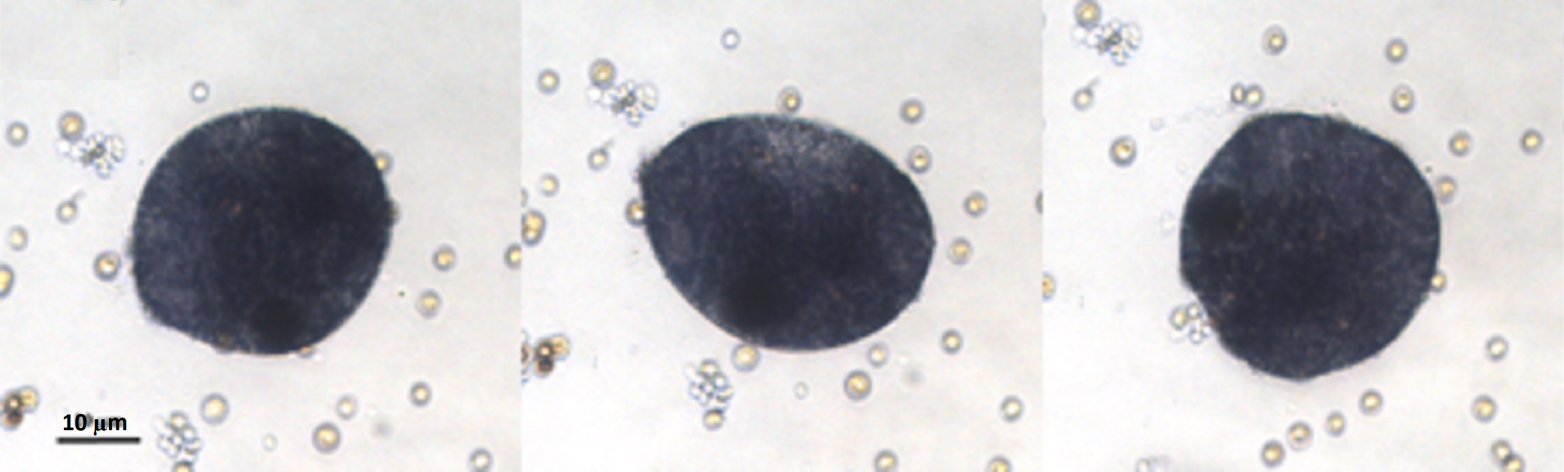
Placozoa bud. Sequence (2 min long) of light micrographs *in vivo* of a bud generated by an organism treated with EtOH. Sequence of light micrographs *in vivo* of a single sphere generated by an organism treated with EtOH. Pictures showing changes in the bud body shape, were taken at the 30th day, when the budding process was in progress. The entire process, from the first recognizable primordium of the small spheres to the detachment, took about 8 h.

On the contrary, the P+EtOH and CTRL groups showed a lower growth velocity; in fact, at the end of the experimental period, these cultures had not yet reached the flex point. Although the logistic parameters were not significantly different from CTRLs, neverthelss after 32 days of growth the P+EtOH group showed a value higher than expected (asterisk in Fig. 1). *In vivo* observations under light microscope of the three groups allowed detecting their reproductive modalities. The CTRLs reproduced only by binary scission, whereas in both treated groups (EtOH and P+EtOH) in addition to binary scission, budding from the dorsal surface of small (30–40 µm) spheres, which remained attached to the bottom of the culture dish, was observed (Fig. 2). Budding process started around the 21st day and persisted for two weeks (until 36th day) in the EtOH group and one week (until 29th day) in the P+EtOH group. The small spheres showed a stage of transformation, which lasted about a week and led them to acquire the flattened form, typical of *T. adhaerens*. These newly formed animals maintained the flattened shape in the EtOH group, whereas those of the P+EtOH group became globose (see next section). At the end of the experiment we decided to maintain the cultures alive as long as possible to evaluate treatment effects over the long term. Notably, all EtOH organisms were found dead at the 51st day, whereas all P+EtOH specimens survived and, rather, regained the typical morphology.

### Effects of *P. spinosa* fruit ethanol extract on *T. adhaerens* phenotype

**Figure 3:**
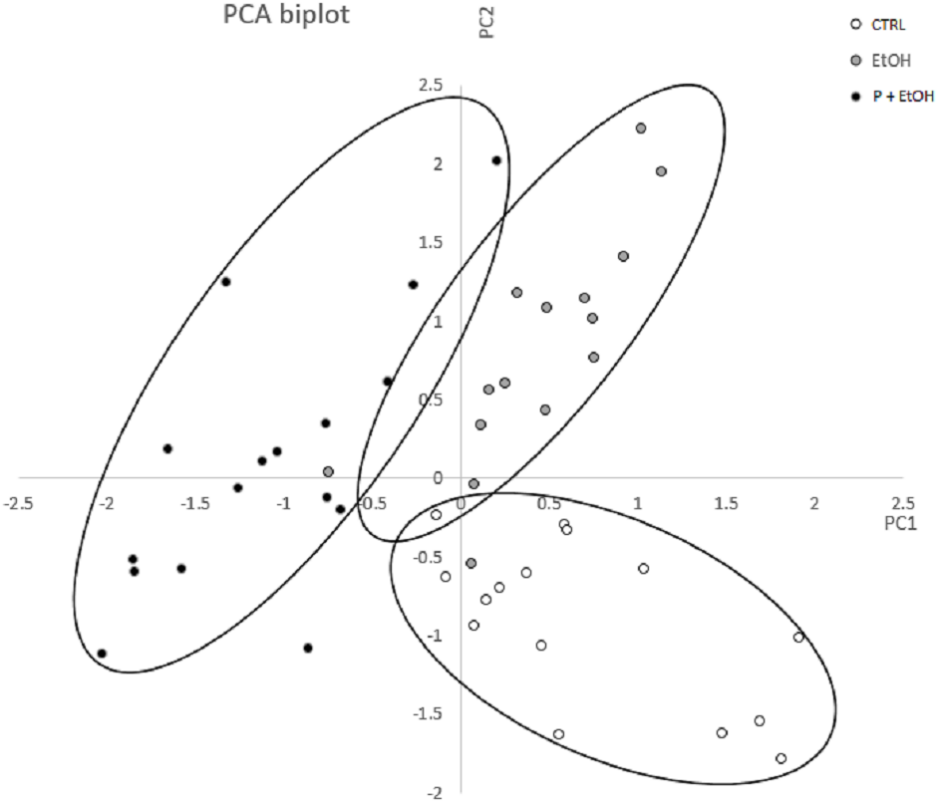
Principal Component Analysis (PCA) represented by a scatter plot of PC1 and PC2. The different groups (CTRL, EtOH and P+EtOH) are displayed with different colours (see legend inside the figure). The 95 % confidence ellipses are also shown. Principal Component Analysis (PCA) represented by a scatter plot of PC1 (highly correlated with Perimeter, Major axis of the best fitting ellipse, Feret’s diameter and Integrated Density) and PC2 (highly correlated with Minor axis of the best fitting ellipse). The different groups (CTRL, EtOH and P+EtOH) are marked differently (black, white and grey; see legend inside the figure). The 95% confidence ellipses are also shown.

A morphometric analysis of the living placozoans was performed, considering a complete set of variables describing shape and size of the organisms, by ImageJ. Values obtained from organisms belonging to each group were analysed by Principal Component Analysis (PCA) to identify and characterize the most significant variables discriminating among the groups. In Fig. 3 is reported the diagram plotted in the bi-dimensional space, defined by the 1st (PC1) and 2nd (PC2) Principal Component functions. As shown, although PC1 and PC2 explain only for the 62.02% of the total variance, which could be considered a moderate result, the three groups are clearly distinguishable in the graph.

By the analysis of the component coefficients, it has been possible to establish that the 1st Principal Component was highly correlated with Perimeter, Major axis of the best fitting ellipse, Feret’s diameter (defined as the longest distance between any two points set along the boundary), and Integrated Density (defined as the sum of the values of the pixels in the image, equivalent to the product of Area and Mean Gray Value, https://imagej.nih.gov/ij/features.html), while the 2nd Principal Component was highly correlated with Minor axis of the best fitting ellipse. This analysis showed that, with respect to the CTRL group, in the EtOH group the Minor axis of the best fitting ellipse (PC2) was significantly different (*p* ≤ 0.05), which suggests a change in dimension; whereas in the P+EtOH group both 1st and 2nd associated parameters were significantly modified (*p* ≤ 0.05), thus revealing that organisms treated with *P. spinosa* fruit ethanol extract (P+EtOH) were different not only in body shape but also in colour.

**Figure 4:**
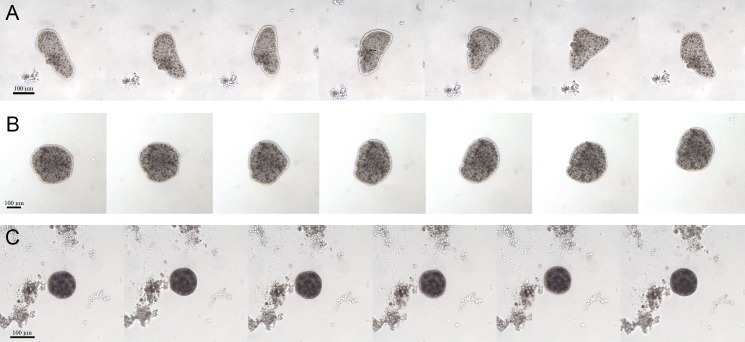
Changes of body shapes. Sequence of images showing the changes of body shapes in CTRL (A), EtOH (B) and P+EtOH (C) experimental groups. All animals were taken from the cultures and photographed at the end of the experiment. Controls showed large and flattened bodies, irregular margins and evident changes in the body shape. Ethanol treated animals showed dark and flattened smaller bodies, regular margins and small changes in the body shape. *P. spinosa* + Ethanol treated animals showed very dark, globose bodies with small diameter and regular margins, without any changes in the body shape.

For the whole time of the observation, the CTRL group had the typical appearance: large and flattened bodies with irregular margins, fast movements (mainly crawling) and rapid changes in shape (Fig. 4A). On the contrary, after about 7 days from the starting of the experiment, the EtOH and P+EtOH organisms showed a decrease in size and altered body morphology. In particular, the EtOH group showed darker and flattened bodies with regular margins, smaller in size than the controls, with slow rotation movements and body shape changes less evident than in CTRLs (Fig. 4B); the P+EtOH organisms showed very dark, small diameter, globose bodies with regular margins, very slow rotations and no morphology changes (Fig. 4C). They spent most of their time standing still and, in this condition, it was easy to observe (Video S1, Fig. 1S) the presence of some short cilia with rounded extremities and of long marginal cilia (maybe used to capture the food). The specimens often stopped, waved their long marginal cilia and then returned to rotate slowly. The phenotypic changes described above were observed in all animals of both groups (EtOH and P+EtOH) until the end of the experiment.

We used optical and electron (TEM, SEM) microscopy to deeply investigate the phenotypes found.

**Figure 5:**
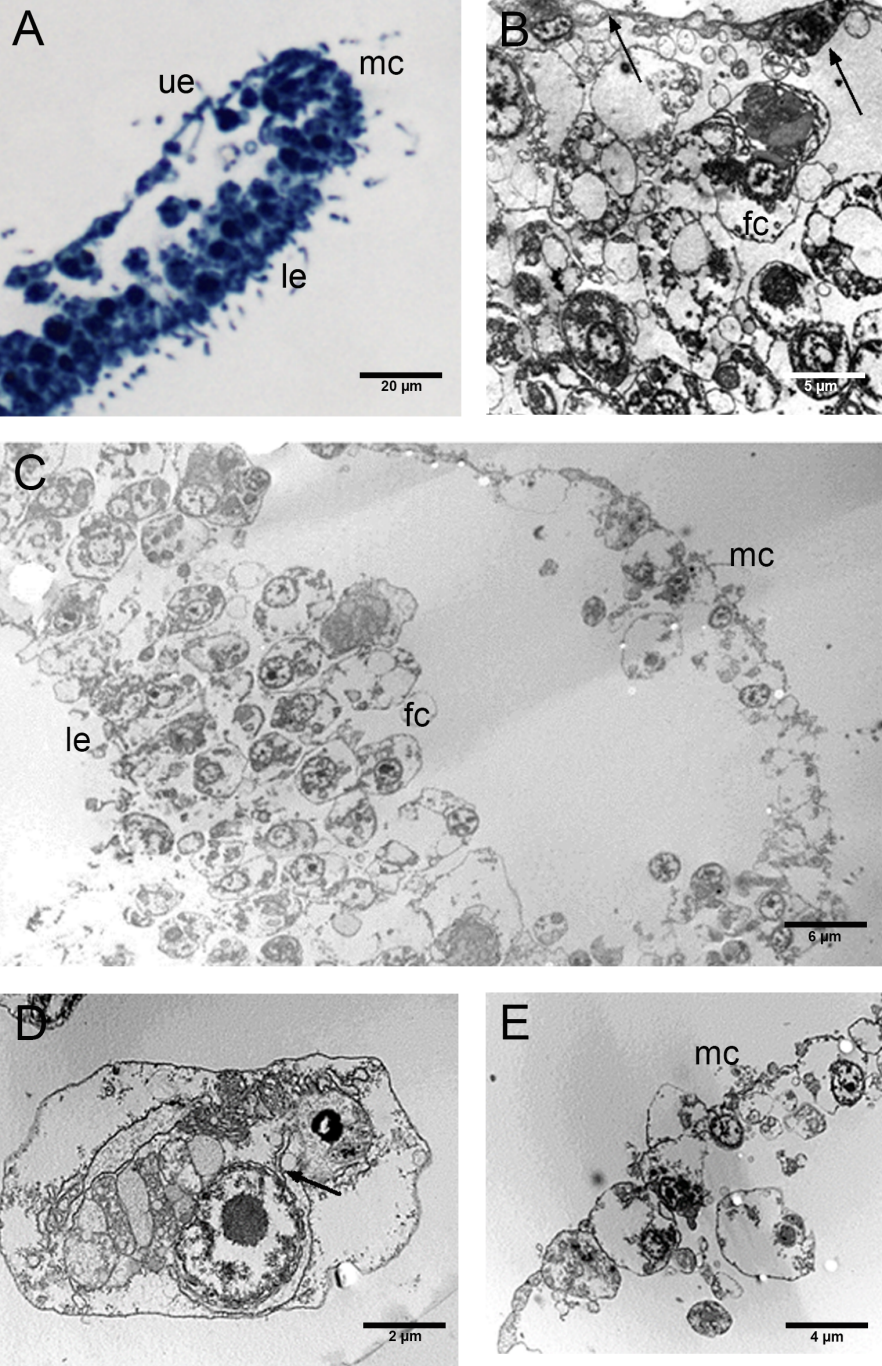
Fine morphology of CTRL Placozoa. (A) Body cross-section showing the upper and lower epithelium, and the margin (optical microscopy). (B) Cross-section of the upper epithelium and fiber cell layers. Arrows indicate flattened cells (T-cells) of the upper epithelium. (C) Body cross-section showing the marginal cells, the fiber cells and the lower epithelium. (D) A single fiber cell showing the cytoplasmic organelles. The continuity of the external nuclear membrane with cisternae of RER is shown (see arrow). (E) Detail of the margin showing the peculiar small cells. (A): Optical microscopy. (B)–(E): TEM microscopy. fc, fiber cells; le, lower epithelium; mc, marginal cells; ue, upper epithelium.

The CTRL group, observed by optical and TEM microscopy, revealed an upper epithelium formed by a thin layer of flat and flagellated cells (T-cells) with a characteristic depression of the soma and two long and thin cytoplasmic prolongations. The depression protruded inside the animal body harbouring the nucleus (Figs. 5A, 5B). The lower epithelium consisted of flagellated, cylindrical cells and few scattered flagellated gland cells (Fig. 5C). Between upper and lower epithelium, there were numerous star-shaped fiber cells arranged in layers and connected to each other forming a three-dimensional syncytium (Fig. 5B). In their cytoplasm, we observed a single large electron-dense vesicle, the mitochondrial complex and several rough endoplasmic reticulum (RER) cisternae (Fig. 5D). These latters are in continuity with the external nuclear membrane, as shown on the right side of the photo (Fig. 5D, see arrow).

A marginal thick cord made up of peculiar ovoidal, remarkably small cells (about 2 µm in diameter) ran around the entire margin of the body (Figs. 5C, 5E).

**Figure 6:**
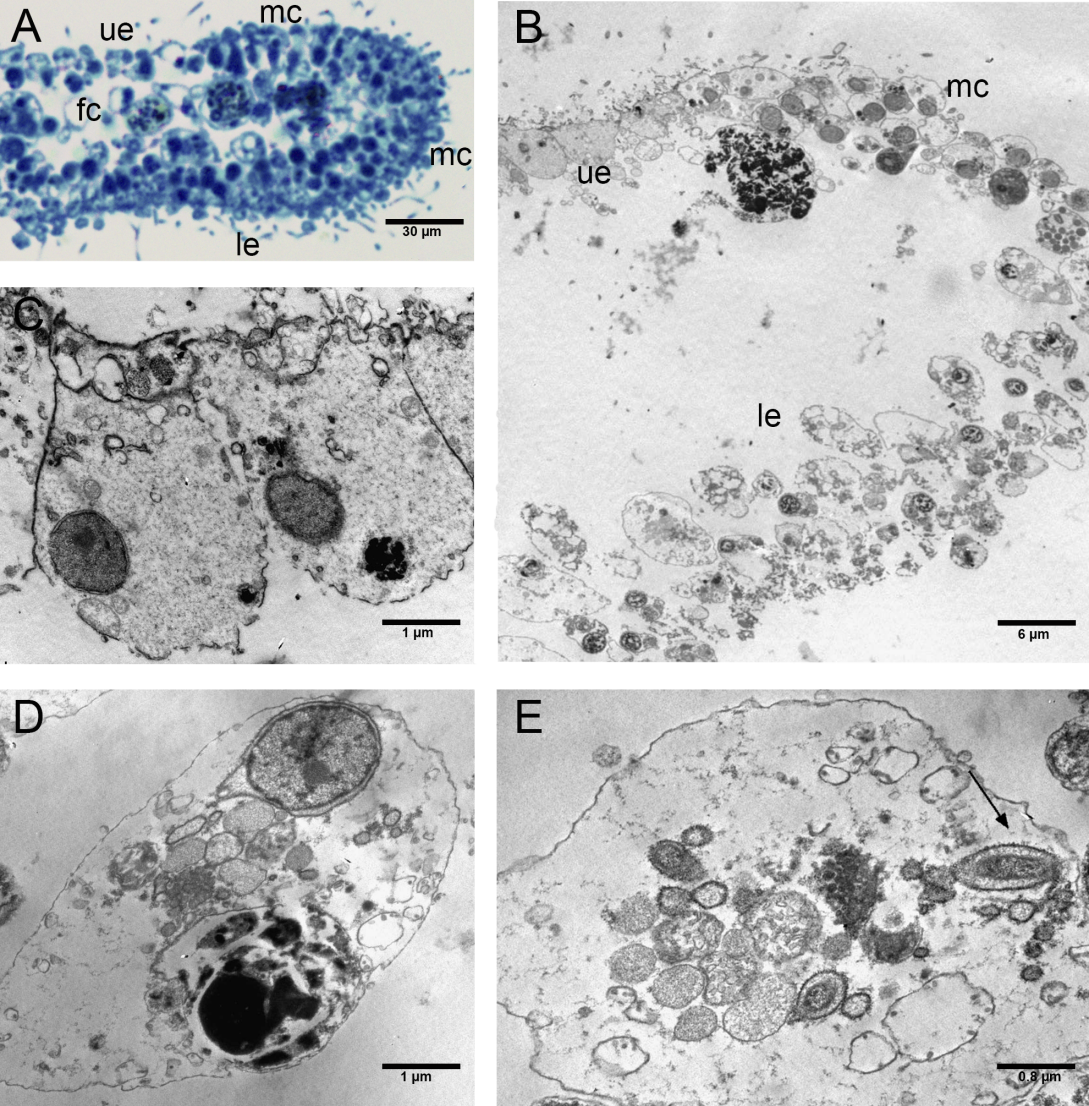
Fine morphology of EtOH Placozoa. (A) Body cross-section showing the upper and lower epithelium, the margin and the inner fiber cells. Note the considerable width of the margin (optical microscopy). (B) Body cross-section showing the upper and the lower epithelium and the thick margin composed of a group of small cells. (C) Detail of two columnar cells of the upper epithelium. (D) Single fiber cell of the inner three-dimensional syncytium showing typical organelles. (E) Fiber cells: note one bacterium (arrow) inside the cisternae of the rough endoplasmic reticulum. (A): optical microscopy. (B)–(E): TEM microscopy. fc, fiber cells; le, lower epithelium; mc, marginal cells; ue, upper epithelium.

The EtOH group had an upper epithelium formed by columnar cells tightly adhering to each other (Figs. 6A–6C). Overall, the lower epithelium appeared similar to that observed in CTRLs, made of cylindrical and gland cells. On the other hand, the marginal cells marked a clear passage between the cells of the upper and lower epithelium (Figs. 6A, 6B). The inner three-dimensional syncytium showed the same organelles observed in the controls (Fig. 6A, 6D). The external nuclear membrane was connected with the cisternae of the rough endoplasmic reticulum where encased bacteria were observed (see Fig. 6E).

**Figure 7:**
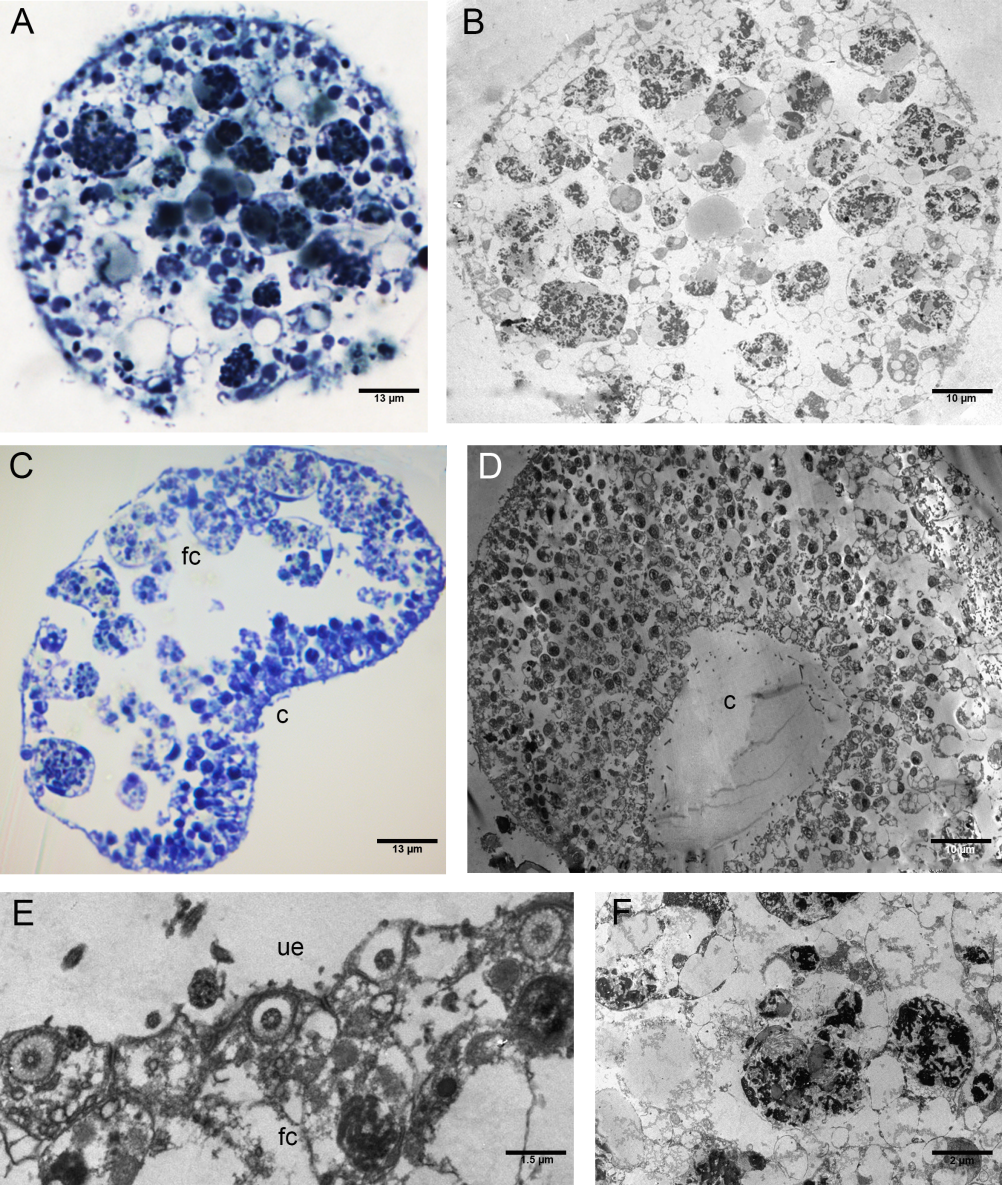
Fine morphology of P+EtOH Placozoa. Frontal semithin (A) and thin (B) section showing the rounded shape and regular margins of the body. Semithin (C) and thin (D) sections showing the cavity formed by lifting of the lower epithelium in the central part of the body. The majority of the fiber cells are located close to the upper epithelium. (E) Detail of the upper epithelium in which the small cuboidal cells are clearly visible. (F) Thin sections of fiber cells: weakly electron-dense lipid granules and strongly electron-dense proteinaceous material are visible. (A), (C): optical microscopy. (B), (D), (E), (F): TEM microscopy. fc, fiber cells; c, cavity; ue, upper epithelium.

**Figure 8:**
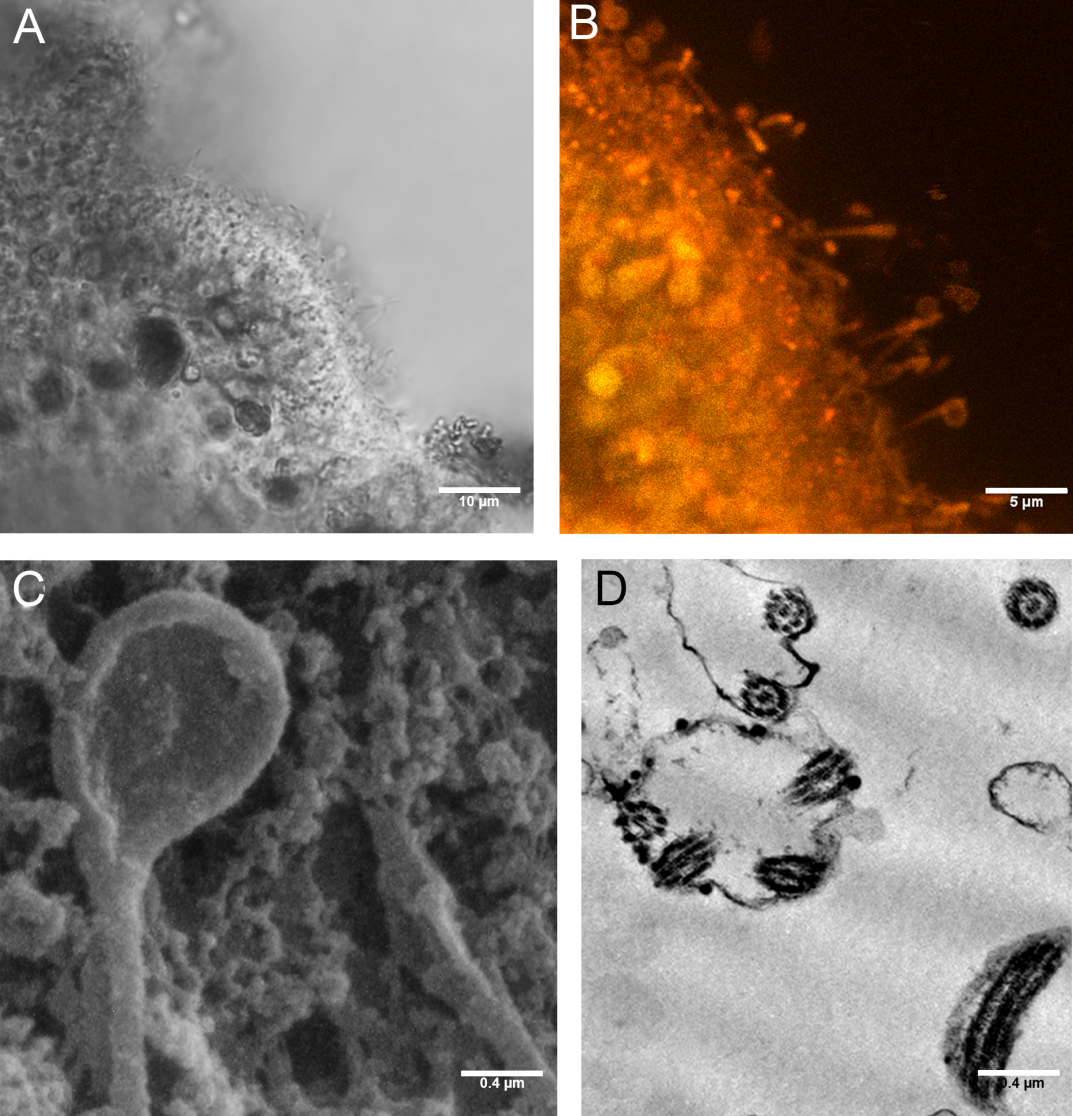
Cilia with rounded extremity in placozoans treated with P+EtOH. Confocal Laser Microscopy: (A) optical section and (B) section showing overlay of Nile Red yellow and red fluorescence. (C) Cilia with the appearance of a teaspoon (SEM Microscopy). (D) Thin section of cilia rounded extremity (TEM Microscopy).

The P+EtOH organisms had a globose shape and regular body margins as showed by frontal semithin and thin sections made close to the upper epithelium (Figs. 7A, 7B). Instead, dorso-ventral semithin sections of the organisms showed the lower epithelium outlining a well evident digestive cavity formed by lifting of the central part of the body (Fig. 7C), which was confirmed also by thin sections (Fig. 7D). The upper epithelium appeared formed by small cuboidal cells tightly adhering to each other (Fig. 7E), while no morphological changes were observed in the lower epithelium. The fiber cells, mainly located close to upper epithelium, showed a cytoplasm rich in brown-yellow lipidic droplets and numerous other proteinaceous inclusions (stained in intense blue) forming large agglomerates, as documented in both semithin and thin sections (Fig. 7F). Notably, only in this experimental group, cilia of the marginal cells showing rounded extremities were observed *in vivo* either under confocal laser microscope (Figs. 8A, 8B), or SEM and TEM.

SEM observations revealed teaspoon-like cilia (Fig. 8C), whereas TEM clearly documented a unique axoneme, folded and forming a loop in the distal portion, surrounded by the cytoplasmic membrane (Fig. 8D).

## Discussion

The current study investigated, for the first time, the effects of *P. spinosa* fruit ethanol extract on reproduction, phenotypic plasticity and feeding behaviour of *T. adhaerens* cultures during a 46 day experimental period. Obtained results showed significant differences when comparing the treated group (P+EtOH) with controls.

### *P. spinosa* ethanol extract effects on *T. adhaerens* reproduction

In P+EtOH we observed a period of excess (i.e., higher than expected) of clone growth after 32 days which was most likely related to the two different reproductive modalities and, particularly, to budding. This asexual reproduction produced small spheres resembling in size and in non-floating behavior the spherical stages formerly reported as “swarmers” (Grell & Benwitz, 1971) or “swarmers-like spheres” (Thiemann & Ruthmann, 1991). The same kind of budding occurred also in EtOH group but for a longer period. The longer duration of the budding in EtOH group with respect to P+EtOH (two weeks vs. one week) may justify the higher number of individuals observed in the first group. Taking into account that: (1) such a type of reproduction, generally rare in laboratory cultures, occurs when environmental conditions become unfavourable (Thiemann & Ruthmann, 1988; Thiemann & Ruthmann, 1991); (2) budding period was longer in EtOH group; (3) treatment with ethanol caused the death of all EtOH specimens after the end of the experiment; (4) P+EtOH organisms showed a shorter budding period and survived long after the end of the experiment; all these items seem to suggest a protective effect of *P. spinosa* ethanol extract. As already reported in available literature, the major phytochemicals found in *P. spinosa* are anthocyanins, common plant pigments able to control different enzymatic activities, to influence several biological mechanisms and modify gene expressions through nuclear receptors (for details see Deineka, Deineka & Sirotin, 2005; Liu et al., 2017; Ya et al., 2018). In particular, it is widely known that anthocyanins protect against damage caused by ethanol: the administration of *Ribes nigrum* (black-currant) juice is able to protect the liver, the brain and the serum of the rats from EtOH induced oxidative stress (Ambrozewicz et al., 2013). The four main pigments, i.e., delphinidin 3-O-glucoside, delphinidin 3-O-rutinoside, cyanidin-3-O-glucoside and cyanidin-3-O-rutinoside contribute up to 97% of the total anthocyanin in black currants. The alanine transaminase and aspartate transaminase values, biomarkers of liver damage, come back to normal after administration of black-currant juice to EtOH-stressed animals. In addition, the expression of PPARα, AMPK, TNF-α, and NFκB confirmed the protective effect of the juice rich in anthocyanins (Ambrozewicz et al., 2013). Lucero Lopez et al. (2011) have evaluated the effect of a diet based on *Amaranthus hypochondriacus* seeds on oxidative stress and antioxidant status in the liver of rats sub-chronically exposed to ethanol concluding that *A. hypochondriacus* is a good source of total phenols such as anthocyanins and exerts a protective effect in serum and in liver of rats intoxicated with ethanol. Anthocyanins, as antioxidants, seem to protect from alcoholic damage, but might be pro-oxidants under certain conditions (Cai et al., 2018). For example, anthocyanins extracted from 12 types of violet sweet potatoes rich in cyanidin derivatives showed a high degree of protection in mice treated with ethanol, however high doses of anthocyanin extract were pro-oxidant to animals as shown by an increase of malondialdehyde and reduction of the level of glutathione (GSH); this could be due to the cyanidin derivatives with an ortho-hydroxyl structure on B-ring (Cai et al., 2018).

### *P. spinosa* ethanol extract effects on *T. adhaerens* phenotype

Relevant morphological differences were revealed by PCA including a complete set of variables describing shape and size of the organisms. The diagram highlighted a clear difference of the organisms in the three different experimental conditions with CTRLs showing the largest size of the body followed by EtOH and P+EtOH groups. This latter also revealed a higher colour density, probably due to a higher content of lipid droplets and protein inclusions in the cytoplasm of the fiber cells, as shown by TEM pictures.

Under electron microscopy (SEM and TEM) the most evident differences among experimental groups were the modifications of the upper epithelium, the content in fiber cells, the occurrence of a digestive cavity in the central part of the animal body as well as of teaspoon-like cilia (visible in confocal and electron microscopy).

In CTRLs, cells of the upper epithelim were T-shaped, which is the normal condition (Guidi et al., 2011; Smith et al., 2014), whereas either in EtOH or P+EtOH they lacked the cytoplasmic prolongations reducing their size and becoming columnar and cuboidal in shape. Such a modification of the upper epithelim justified the variations of the organisms both in terms of phenotype (i.e., body shape ranging from irregular (CTRLs) to rounded (EtOH) and globose (P+EtOH)) and dimension (i.e., a progressive decrease in diameter from CTRLs to P+EtOH). Furthermore, from an ultrastructural point of view, the fiber cells of all specimens showed the typical cytoplasmic organelles with the exception of the high content of lipid granules and proteinaceous material in the P+EtOH, maybe related to the blackthorn extract supply. In this condition, also a different (i.e., closer to the upper epithelium) position of the fiber cells was recorded suggesting a transepithelial cytophagy carried out through the upper epithelium in addition to the external digestion within the digestive cavity (Wenderoth, 1986; Smith, Pivovarova & Reese, 2015).

It is noteworthy that very clear marginal cells were observed in the placozoans of all the three experimental subcultures in agreement with Guidi et al. (2011) and in contrast with Smith et al. (2014) who did not describe them underestimating the number of the cell types and misinterpreting the structural plan of *Trichoplax* body (Schierwater & Eitel, 2015).

### Feeding behaviour of *T. adhaerens*

A different feeding behaviour was observed in the various treatments. In particular, CTRLs showed the typical feeding behaviour (Schierwater, 2013; Schierwater & Eitel, 2015): moving fast around, crawling with rapid changes in shape and, as already reported by Smith, Pivovarova & Reese (2015), spending much of the time in various movements on the substrate. In contrast, P+EtOH placozoans rotated very slowly without changes in their shape. They stayed all the time in the same position by means of the teaspoon-like cilia at the margins of the body. These structures, observed for the first time, may be regarded as adhesive organs; on the contrary, the long cilia of the upper epithelium moved quickly, probably (in our opinion) to attract food particles, subsequently captured by transepithelial cytophagy by the fiber cells. P+EtOH organisms presented also a “permanent” digestive cavity observed *in vivo* from the 7th day until the end of the experiment, clearly visible in both semithin and ultrathin sections, which was likely a morphological adaptation to the abundant food supply. On the other hand, EtOH organisms showed a feeding behaviour similar to the CTRLs (temporary digestive cavity), but with slower movements, probably related to the phenotypic changes (i.e., smaller size and rounded shape). Phenotypic variations observed in both EtOH and P+EtOH organisms were probably due to the action exerted by ethanol. As recently reported, Von der Chevallerie, Rolfes & Schierwater (2014) observed changes in phenotype after addition of nutlin-3 and roscovitine inhibitors of p53/Mdm2. The authors documented stronger phenotypic variations after inhibitor treatment providing strong evidence for a disturbance of the *T. adhaerens* development likely due to an imbalance in the control of central to marginal tissue growth ratio that, in Placozoa, is normally reported as tightly regulated. Thus, the inhibitor treatment seemed to influence the stem cell proliferation and led to an enlargement of body margins since placozoans stem cells are known to be located in this area and control both growth and division rate (Jakob et al., 2004). In agreement with these observations, it is possible to hypothesize a different growth rate between central and marginal tissues in our cultures, especially in EtOH organisms, where a marginal thicker cord was clearly detected. Thus, the different growth rate of these tissues may explain also the abnormal phenotypes found.

## Conclusions

For the first time, *T. adhaerens* cultures were used as model system to evaluate *in vivo* effects of natural extracts. Particularly, in the present study *P. spinosa* ethanol extract bioactivity was assessed. Obtained results confirmed our working hypothesis regarding a protective activity of the extract, most likely due to the presence of active phytocomponents, in particular the anthocyanins. In fact, optical and confocal along with SEM and TEM observations of treated (P+EtOH) organisms allowed to highlight significant differences in morphology and reproduction modalities compared to controls. At the moment, it seems reasonable to conclude that blackthorn ethanol extract, used as food additive, modulates ethanol induced abnormalities (including morphology changes, different reproduction modalities and reduced viability shown by EtOH organisms), potentially indicating a beneficial effect. Since, in this first preliminary work, the study was carried out using the whole *P. spinosa* ethanol extract, at the moment, we are not able to indicate with certainty which components are mainly responsible for the protective action. This aspect will be explored, in a second series of experiments, using different fractions of the extract in order to identify the active ones.

A better characterization of the properties of *P. spinosa* ethanol extract by *in vitro* and *in vivo* models, will be of interest to promote new bioactive compounds in nutraceutical preparations to be used as food supplements.

##  Supplemental Information

10.7717/peerj.6789/supp-1 Figure S1 Growth curves Non linear logistic regression growth curve.
*X* axis: culture days; *Y* axis: Placozoa number.

10.7717/peerj.6789/supp-2 Figure S3 PCA analysis

10.7717/peerj.6789/supp-3 Figure S4 Optical pictures of representative numbers of animals fromall experimental groups.

10.7717/peerj.6789/supp-4 Supplemental Information 1 The video showing feeding behaviour The video shows the feeding behaviour of a living P+EtOH treated specimen: the long marginal cilia are in active movement whereas the short ones, with rounded extremities, move slowly.

10.7717/peerj.6789/supp-5 Supplemental Information 2 Shortcilium

## Additional Information and Declarations

### Data Availability

The following information was supplied regarding data availability: The raw data is available as Supplemental Files.
