# Supplementary figures and images for: Bioeffects of Prunus spinosa L. fruit ethanol extract on reproduction and phenotypic plasticity of Trichoplax adhaerens Schulze, 1883 (Placozoa)
Source: PeerJ. 2019 Apr 18;7:e6789. doi: 10.7717/peerj.6789 (PMC6475577; doi:10.7717/peerj.6789)

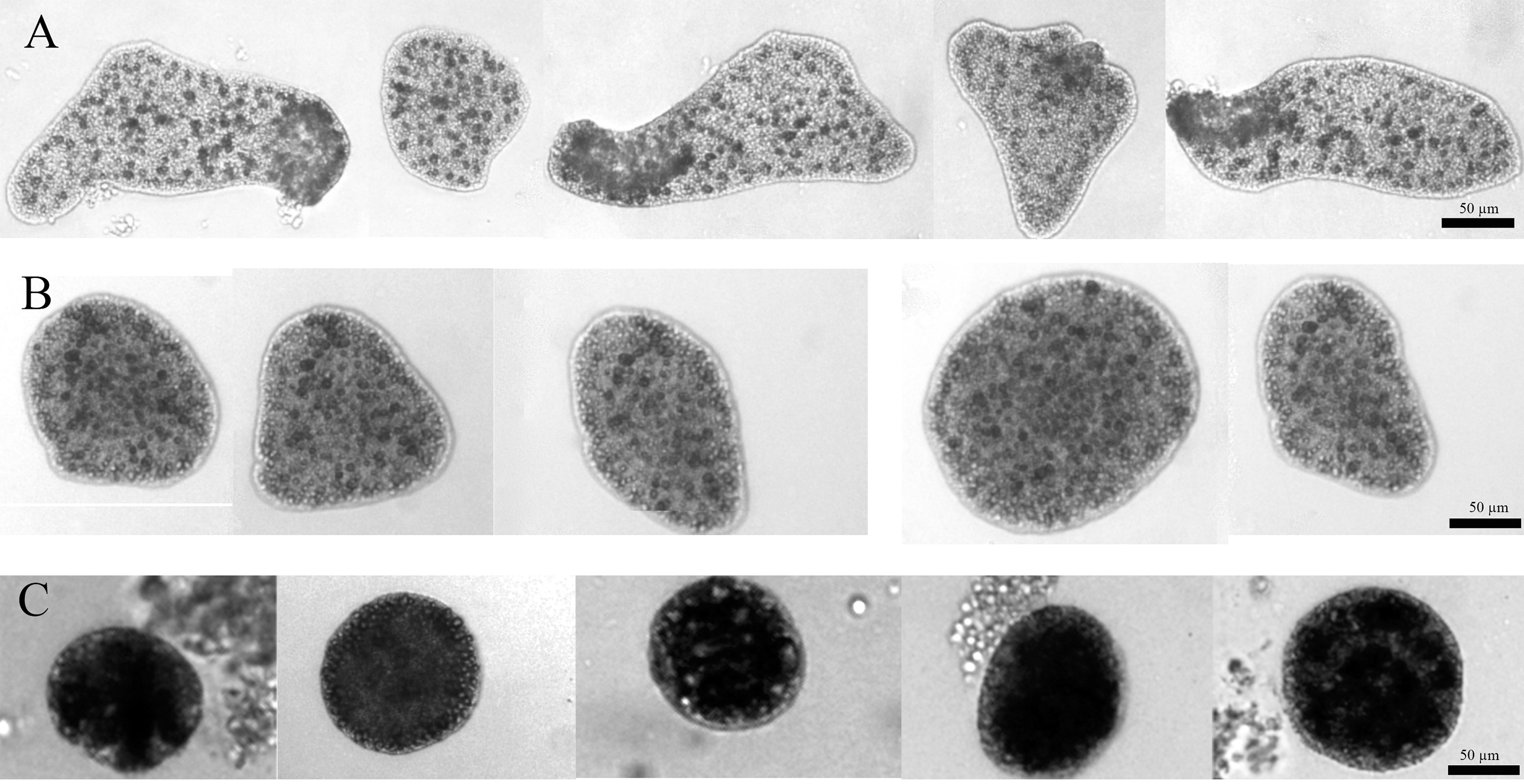

Supplement: Figure S4 [file peerj-07-6789-s003.png]

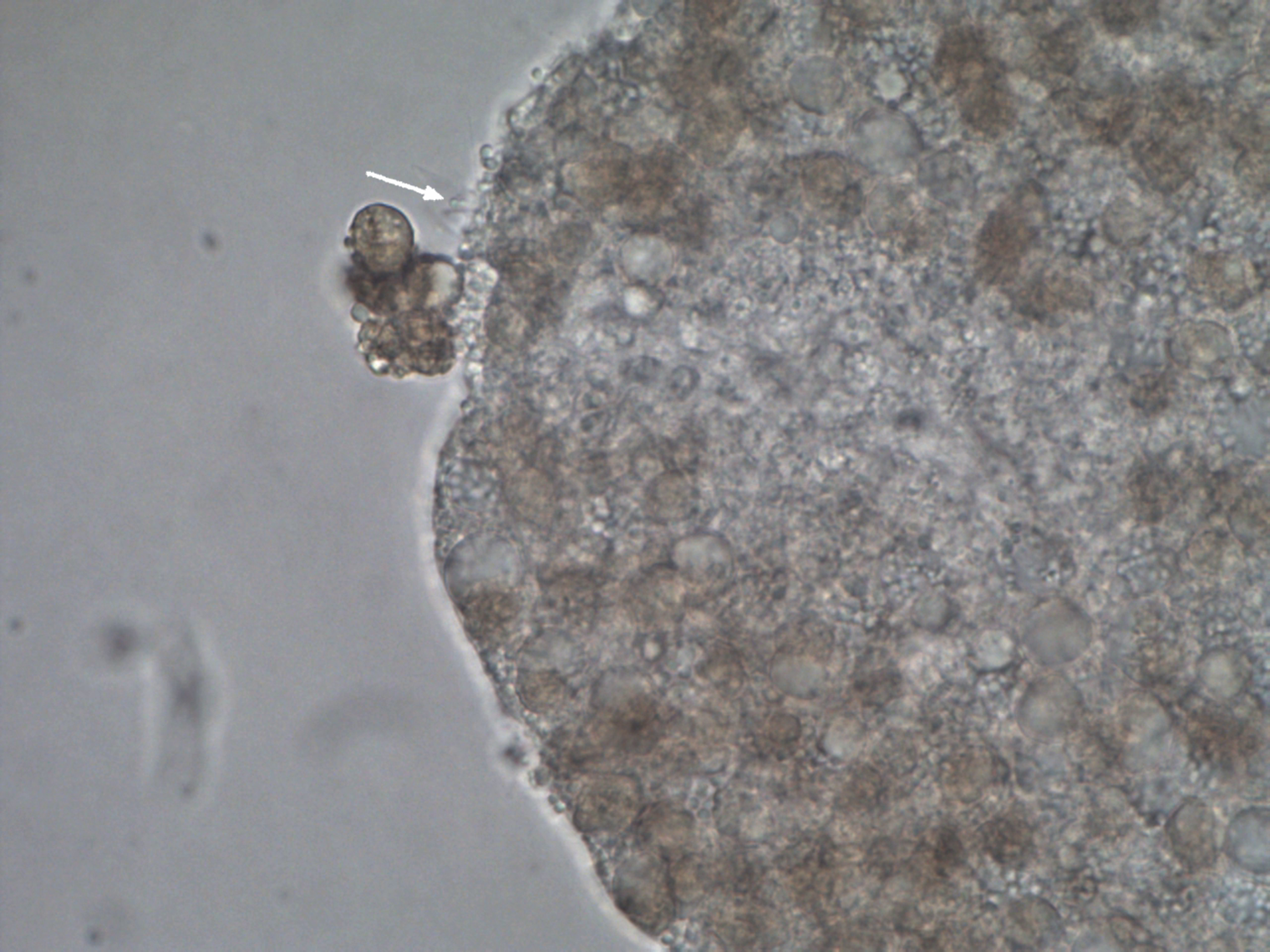

Supplement: Supplemental Information 2 [file peerj-07-6789-s005.png]
